# Electromyogram synergy control of a dexterous artificial hand to unscrew and screw objects

**DOI:** 10.1186/1743-0003-11-41

**Published:** 2014-03-21

**Authors:** Benjamin A Kent, Nareen Karnati, Erik D Engeberg

**Affiliations:** 1 Mechanical Engineering Department, The University of Akron, ASEC Rm. 101, Akron, OH, USA; 2 Biomedical Engineering Department, The University of Akron, ASEC Rm. 275, Akron, OH, USA

**Keywords:** Amputee, Dexterous hand, Electromyogram, Grasp, Prosthetic hand, Sliding mode control, Synergy

## Abstract

**Background:**

Due to their limited dexterity, it is currently not possible to use a commercially available prosthetic hand to unscrew or screw objects without using elbow and shoulder movements. For these tasks, prosthetic hands function like a wrench, which is unnatural and limits their use in tight working environments. Results from timed rotational tasks with human subjects demonstrate the clinical need for increased dexterity of prosthetic hands, and a clinically viable solution to this problem is presented for an anthropomorphic artificial hand.

**Methods:**

Initially, a human hand motion analysis was performed during a rotational task. From these data, human hand synergies were derived and mapped to an anthropomorphic artificial hand. The synergy for the artificial hand is controlled using conventional dual site electromyogram (EMG) signals. These EMG signals were mapped to the developed synergy to control four joints of the dexterous artificial hand simultaneously.

Five limb absent and ten able-bodied test subjects participated in a comparison study to complete a timed rotational task as quickly as possible with their natural hands (except for one subject with a bilateral hand absence), eight commercially available prosthetic hands, and the proposed synergy controller. Each test subject used two to four different artificial hands.

**Results:**

With the able-bodied subjects, the developed synergy controller reduced task completion time by 177% on average. The limb absent subjects completed the task faster on average than with their own prostheses by 46%. There was a statistically significant improvement in task completion time with the synergy controller for three of the four limb absent participants with integrated prostheses, and was not statistically different for the fourth.

**Conclusions:**

The proposed synergy controller reduced average task completion time compared to commercially available prostheses. Additionally, the synergy controller is able to function in a small workspace and requires less physical effort since arm movements are not required. The synergy controller is driven by conventional dual site EMG signals that are commonly used for prosthetic hand control, offering a viable solution for people with an upper limb absence to use a more dexterous artificial hand to screw or unscrew objects.

## Background

The mechanical dexterity of all commercially available prosthetic hands is less than the human hand. Most commercially available prosthetic hands like the Motion Control Hand [1] and the SensorHand Speed [2] have a single degree of freedom (DOF). However, there has recently been a shift toward more dexterous prosthetic hands such as the commercially available i-Limb [3] which has five motors; one to drive each digit. Other new prostheses such as the bebionic hand (RSLSteeper, UK) and the Michelangelo Hand [4] feature four fingers and a thumb and make use of underactuated mechanisms. Despite improvements in mechanical dexterity, clinical practice for EMG control of these devices has remained largely unchanged since the advent of myoelectric control.

Prosthetic hands are often controlled by two EMG signals placed on an antagonistic muscle pair [5]. The signals from these two antagonistic muscle groups are then differenced to produce a dual polarity control signal for the motor of the prosthesis in an open loop or force control scheme [6], allowing control of only one joint or function at a given time. However, the control interface for prostheses has been identified as a potential area of improvement by everyday users [7,8]. To help overcome this problem, many different methods of control and signal processing have been proposed: neural networks [9], machine learning techniques [10], fuzzy clustering [11], and wavelet transforms [12], to name a few [13,14]. There are several problems presented by one or more of these control techniques such as increased time delays to process EMG signals, computationally expensive control algorithms, need of four or more EMG electrodes, increased training time, imperfect EMG pattern recognition, and lack of proportional force control. To date, none of the aforementioned techniques have gained widespread clinical use. To further facilitate the shift towards more mechanically dexterous prosthetic hands, an intuitive and robust control interface is still needed.

One significant hurdle preventing a high level of integration of the artificial hand into the body image of the user is the inability to simultaneously control many DOFs independently, which is an area of improvement desired by prosthesis users [15]. Despite recent developments in direct neural interfaces [16] and techniques such as targeted muscle reinnervation [17], the number of inputs that can be extracted to control a prosthesis is limited. It would be quite difficult to extract twenty independent signals to control the twenty DOFs of a dexterous anthropomorphic hand like the Shadow Hand [18], even with the simultaneous use of these advanced techniques hybridized into one system. The cognitive burden required to coordinate these twenty signals would also be inordinately high. This is because many aspects of control of the human hand occur subcortically [19]. For these reasons, the use of grasp synergies is a beneficial technique because a limited number of control inputs are used to specify the action of a larger number of joints [20-23]. The concept of grasp synergies effectively reduces the dimensionality of dexterous hands by coupling the motions of multiple finger and thumb joints together. People use this control strategy frequently while reaching to grasp different objects [24], when manipulating different objects [25], when using tools [26], and in many other situations [21].

Grasp synergies have great implications for upper limb prosthetics, as it allows for driving complex motions from a limited input set, reducing the cognitive burden of the operator. Thus, the difficult problem of controlling dexterous manipulators such as the ACT or Shadow hands can be simplified [18,27]. Brown and Asada used a principal component analysis (PCA) and a mechanical implementation to control a 17-DOF (10 active) hand via two control inputs [28]. A similar approach was undertaken by Xu and colleagues with an underactuated prosthetic hand for a manipulation task [29]. Matrone *et al*., recently proposed a PCA based control algorithm to implement postural synergies on an underactuated prosthetic hand also using two inputs [30]. Likewise, grasp synergies have been used to increase the information throughput for brain machine interfaces: a 10 DOF virtual hand was controlled by two electrocorticographic signal recording electrodes [31]. Whether it is a mechanical or control based implementation, grasp synergies have shown great promise in creating more anthropomorphic motions of dexterous hands while requiring a low number of control inputs.

With currently available prosthetic hands, upper-limb prosthesis users have a diminished ability to do simple things like catch an object, put on a tie, unscrew a bottle cap, or drive a car (among many other activities) [32]. These are highly significant problems for those with a hand absence [7,8,15,32,33]. An intelligent, dexterous prosthesis could substantially improve these problems.

**Figure 1:**
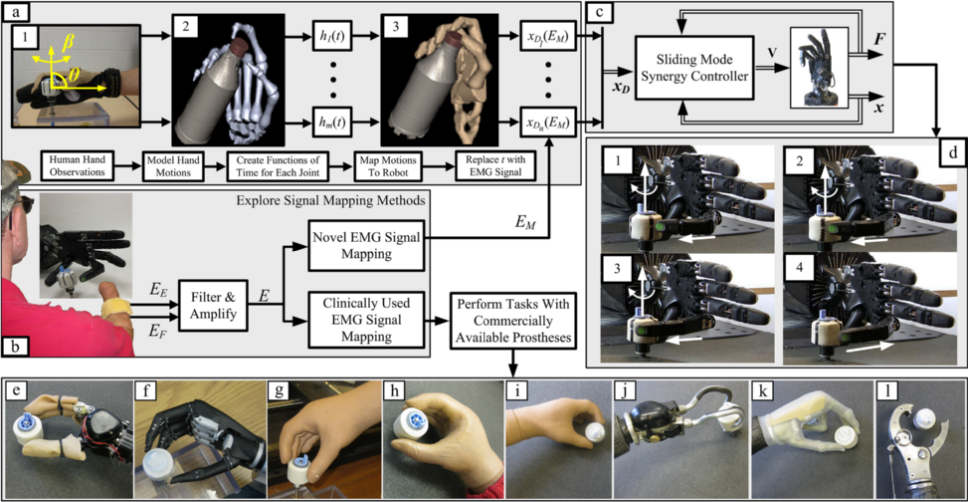
**Methods overview. (a)** A synergy was developed from the recorded human joint motions of ten test subjects while unscrewing and screwing a bottle cap. These motions were approximated by a family of sinusoids which share a common frequency of oscillation. **(b)** The EMG signals of 15 test subjects were mapped to the control input *E*_*M*_ used to drive the synergy controller. Two EMG mapping methods were considered: Rhythmic and Threshold. The performances of these EMG mapping methods were compared to a traditional control scheme and system. **(c)** The synergy controller was implemented on the C6M Dexterous Shadow Hand through a sliding mode controller using joint angle and tendon force feedback. **(d)** The synergy controller was evaluated by 10 able-bodied (1-10) and five limb absent test subjects (A1-A5). The performance of the proposed technique was compared to eight different prosthetic hands in total. **(e)** An able-bodied test subject performing the unscrewing task with the Motion Control Hand (MC) under sliding mode control. **(f)** An able-bodied test subject performing the unscrewing task with the i-Limb Ultra Hand (ILU) under open loop control. **(g)** Test subject A1 with his SensorHand Speed (SHS). **(h)** Test subject A2 with one of her MyoHand VariPlus Speeds (VPS). **(i)** Test subject A3 with his MyoHand VariPlus Speed. **(j)** Test subject A4 performed the rotational task with his Motion Control ETD Hook (ETD), **(k)** i-Limb (IL), and **(l)** body-powered Grip Prehensor (BP).

In response to these problems for those with a hand absence, a new synergy was developed to enable unscrewing and screwing motions of a dexterous artificial hand [18] with a single pair of EMG preamplifiers, as are used by myoelectric prosthesis users daily [34]. This synergy design process is outlined as follows: the finger and thumb joint trajectories of able-bodied human test subjects were recorded as they rotated a threaded cap. From this data, a synergy controller was developed which approximated the human motions with sinusoidal joint angle trajectories (Figure 1(a)). This was performed prior to the current study, and the complete details of this portion are provided in Appendix A. The EMG signals of five limb absent and ten able-bodied test subjects were then mapped to a control input (*E*_*M*_) to drive the synergy; two different EMG mapping methods were explored (Figure 1(b)). The sinusoidal synergy was then used to drive the C6M Dexterous Shadow Hand via a sliding mode controller (Figure 1(c)) to screw and unscrew an object (Figure 1(d)). The performance of the synergy controller was then compared to a one DOF prosthesis (Figure 1(e)) and an i-Limb Ultra (Figure 1(f)) operated by ten able-bodied test subjects. Three individuals with transradial amputations and two persons with congenital hand absences also compared the sinusoidal synergy controller for the Shadow Hand to their current prostheses for daily use (Figure 1(g-l)). In total, nine different artificial hands were evaluated by both groups in a timed rotational task. To the best knowledge of the authors, this paper documents the first instance that people with an upper limb absence have had EMG control of a dexterous artificial hand to unscrew and screw any object.

## Materials and methods

### Dual polarity electromyogram control signal

As mentioned previously, most commercially available prosthetic hands use a dual polarity control signal formed from an antagonistic muscle pair [5]. For those users with a transradial disarticulation or deficiency, the extensor digitorum communis (EDC) and flexor carpi radialis (FCR) muscles are commonly used [13,14].

To suitably process the voltages caused by contractions of the muscle groups, they are first filtered, rectified, and amplified. The resulting EMG signals for the extensor muscles (*E*_*E*_) and flexor muscles (*E*_*F*_) are then differenced to produce a dual polarity signal that is convenient to control a motor:

(1) $$E=E_{E}-E_{F}$$

### Artificial hand systems

#### *Motion control hand*

The Motion Control Hand (Figure 1(e)) has a single DOF with the thumb and forefingers connected through a four-bar linkage system. An A1321 Hall effect sensor is used to measure the position (*x*_*M1*_). Strain gauges mounted on the thumb measure the normal force (F_N_).

The Motion Control Hand is controlled with a hybrid force-velocity sliding mode controller which has been described elsewhere [1] and is of the form

(2) $$V_{\mathrm{MC}}=-C_{\mathrm{MC}}\mathrm{sat}\left(S_{\mathrm{MC}}\right).$$

*V*_*MC*_ is the voltage control law, *C*_*MC*_ is a positive constant and *S*_*MC*_ is the sliding manifold comprised of EMG signals (*E*), position (*x*_*M1*_), velocity, and force feedback. The hybrid sliding mode controller is used to improve the control of force and velocity for the prosthesis. See [1] for a stability and robustness analysis of sliding mode control for this particular system.

#### *i-Limb ultra hand*

The i-Limb Ultra (Figure 1(f)) has five actuated DOFs with one motor to control each digit [3]. The i-Limb Ultra does not use sensor feedback so an open-loop controller was implemented on the i-Limb Ultra Hand:

(3) $$V_{\mathrm{IL}}=C_{\mathrm{IL}}E.$$

In (3), *C*_*IL*_ is a constant gain. This technique is commonly used to control current prosthetic hands [6].

#### *The shadow C6M Hand*

**Figure 2:**
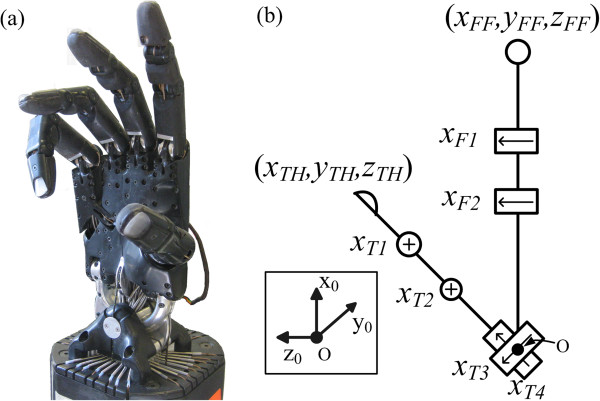
**Hardware overview. (a)** The C6M Shadow Hand has 24 joints and 20 DOFs. **(b)** The kinematic diagram and digit/joint naming convention of the first finger and thumb of the Shadow Hand. The origin is designated by O and the y_0_ axis is into the page. Axes of rotation are visualized as black arrows. Axes of rotation perpendicular to the page are designated by an (X).

The Shadow C6M Hand (Figure 2(a)) is a 24 joint, 20-DOF underactuated tendon-driven anthropomorphic manipulator. However, only two DOFs of the first finger and four DOFs of the thumb are used in the experiments presented in this paper (Figure 2(b)). Strain gauges are used to measure the force in each tendon. Hall effect sensors within the hand provide joint angle data for each joint with a resolution <1°. While the size and mass of the Shadow Hand are currently too large to permit use as a functional prosthesis, it is a useful test bed for control algorithms.

The four DOFs of the thumb used in this paper arise from the carpometacarpal (CMC), metacarpophalangeal (MCP), and interphalangeal (IP) joints. The flexion/extension angle of the IP thumb joint is *x*_*T1*_ and the flexion/extension of the MCP joint of the thumb is *x*_*T2*_. Joints *x*_*T3*_ and *x*_*T4*_ are the angles of the abduction and circumduction of the CMC joint, respectively. The two DOFs of the first finger are the flexion/extension of the MCP (*x*_*F2*_) and proximal interphalangeal (PIP), (*x*_*F1*_) joints (Figure 2(b)).

### Sinusoidal synergy controller

#### *Sinusoidal joint approximations*

To gain inspiration for the synergy controller with the Shadow Hand, a human hand study was initially performed ((Figure 1(a)), described in Appendix A). Using results from this initial human hand study and the process outlined in [18], a sinusoid was used to approximate the human finger and thumb motions for application to the Shadow Hand. The sinusoidal approximation of a joint *k* is given by

(4) $$x_{D_{k}}=A_{k}\sin\left(\mathrm{\omega t}+\phi_{k}\right)+b_{k}$$

where *A*_*k*_ is the amplitude, *ϕ*_*k*_ is the phase offset, and *b*_*k*_ is the angular position offset of sine wave *k*. A sinusoid was generated for two joints in the thumb (*x*_*T1*_ and *x*_*T2*_) and first finger (*x*_*F1*_ and *x*_*F2*_) for the Shadow Hand to approximate the human motions (Table 1). This process entailed first approximating the human joint motions as *m* sinusoidal functions of time (*h*_*1*_(t) – *h*_*m*_(t)), then mapping those functions to *n* joints of the Shadow Hand system $\left(x_{D_{1}}\left(t\right)-x_{D_{n}}\left(t\right)\right)$, (Figure 1(a)).

**Table 1:** Sine wave parameters for shadow hand (rad)

**Joint**	**Phase offset: *ϕ***_***k***_	**Amplitude: *A***_***k***_	**Position offset: *b***_***k***_
*x*_*F1*_	-0.9425	0.635	0.975
*x*_F2_*	1.571	0.319	0.900
Thumb kinematic model			
*x*_*T4*_	0.000	0.000	0.450
*x*_*T3*_	0.000	0.000	1.257
*x*_*T2*_	1.856	0.400	0.000
*x*_*T1*_	1.971	0.490	0.350

^*^Reference Joint.

#### *Fingertip trajectories in Cartesian space*

**Figure 3:**
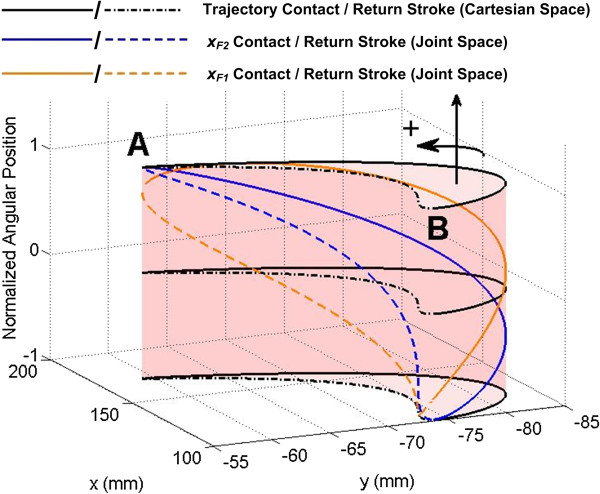
**Fingertip Cartesian space trajectories.** Elliptical trajectory of the index finger in Cartesian space as the finger joints travel along the developed sinusoidal trajectories. The z-axis represents the normalized joint positions of x_F1_ and x_F2_ corresponding to any fingertip location. As these joints move through their sinusoidal trajectories, they create a periodic elliptical motion of the fingertip in Cartesian space, enabling rotation of the object during the contact stroke. The Cartesian locations of the beginning and end of the contact and non-contact strokes are designated by the A and B.

In Cartesian space, the sinusoidal joint trajectories yield elliptical fingertip trajectories that are periodic on 2*π* (Figure 3). For the purposes of the present work, the elliptical trajectories are considered in two halves. While 0 ≤ *t* ≤ *π*, the finger is considered to be in the “contact stroke” of the synergy, where both the first finger and thumb will contact the object, causing rotation. While *π* < *t* < 2*π*, the finger and thumb are in the “return stroke” where no contact or rotational motion of the grasped object occurs. This is clear from Figure 3, where the joint angles required from *x*_*F1*_ and *x*_*F2*_ to produce the Cartesian fingertip position are also displayed.

#### *Shadow hand sinusoidal synergy controller driven by electromyogram*

**Figure 4:**
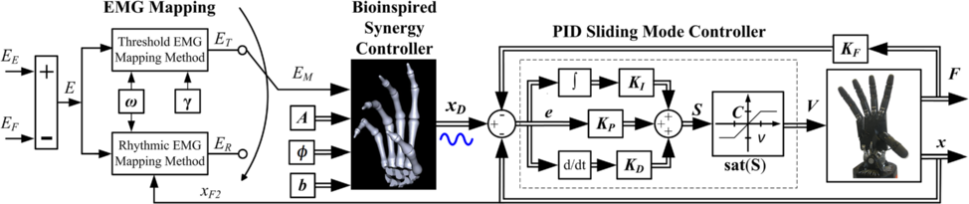
**Sinusoidal synergy controller block diagram.** PID sliding mode control diagram for the sinusoidal synergy controller. A, b, and *ϕ* are the joint amplitudes, position offsets, and phase offsets, of the sinusoids in the controller, respectively. These sinusoids are used to drive each joint of the Shadow Hand individually using only a single EMG input. ν is the slope of the sliding manifold. Two different EMG mapping methods were evaluated by five individuals with a hand absence as the input to the synergy controller, *E*_*M*_. With the Rhythmic EMG mapping method, *E*_*M*_ = *E*_*R*_, and a switching function δ determines whether the EMG signals are mapped to the contact or return strokes of the synergy (8), Figure 3. δ is dependent upon the position of the reference joint, *x*_*F*2_ (Table 1). In the Threshold method, *E*_*M*_ = *E*_*T*_, and when either the EDC or FCR muscles are above a predetermined threshold, γ, *E*_*M*_ within the family of sinusoids (5) is incremented or decremented to produce screwing or unscrewing motions as shown in Figures 6 and 7. The speed of the synergy is constant and determined solely by *ω* for the Threshold method. For the Rhythmic method, the speed of the synergy is affected by *ω* and also by the rate of operators’ muscle contractions.

Because EMG signal amplitude is related to both force/torque and position [35], the EMG synergy controller is implemented within a hybrid position/force controller that employs tendon force feedback (Figure 4).

The developed sinusoidal synergy controller is of the form

(5) $$x_{D}=\left[\begin{array}{ccc} A_{1} & \cdots & 0\\ \vdots & \ddots & \vdots \\ 0 & \cdots & A_{n} \end{array}\right]\;\left[\begin{array}{c} \sin\left(E_{M}+\phi_{1}\right)\\ \vdots \\ \sin\left(E_{M}+\phi_{n}\right) \end{array}\right]+\left[\begin{array}{c} b_{1}\\ \vdots \\ b_{n} \end{array}\right]$$

where ***x***_***D***_ ∈ ℝ^*nx*1^ (*n* = 6) is the vector of desired joint angles. The amplitude (*A* ∈ ℝ^*nxn*^), phase shift (*ϕ* ∈ ℝ^*nx*1^) and joint angle offset (*b* ∈ ℝ^*nx*1^) are determined from the observations of the human data and are included in Table 1. As shown in Figure 1(a,b), the time vector in (4) is replaced by EMG signals (*E*_*M*_) in (5) to control the synergy. Controlling the synergy in this manner simplifies the problem greatly since only one input, *E*_*M*_, is needed to produce coordinated, temporally synchronized motions.

Of the six joints used in the controller, four vary sinusoidally while *x*_*T3*_ and *x*_*T4*_ are constant and used solely to properly position the thumb relative to the first finger. With a passive thumb circumduction joint (as is currently used with the i-Limb and bebionic hands [36]), this positioning could be achieved manually, and the number of active joints would be reduced to four.

To facilitate sliding mode control of the Shadow Hand, an error term is defined as

(6) $$e=x_{D}-x-\left[\begin{array}{ccc} K_{1} & \cdots & 0\\ \vdots & \ddots & \vdots \\ 0 & \cdots & K_{n} \end{array}\right]\;\left[\begin{array}{c} F_{1}\\ \vdots \\ F_{n} \end{array}\right]$$

Here, *F*_*k*_ and *K*_*k*_ are the measured tendon force and the corresponding gain for any joint *k*. Inclusion of tendon force feedback in the control law is another significant difference in this paper compared to prior work [18,34]. This has been done to improve the transition from the noncontact to contact states of operation inherently required while rotating an object as in Figure 1(d). The end result is that faster operational speeds are enabled through the inclusion of tendon force feedback because it increases the compliance of the closed loop system. This technique is beneficial because it facilitates both the control of position along the path of the synergy and the forces applied by the Shadow Hand through a single EMG input. A conceptually similar hybrid control ideology has been used for the Motion Control Hand (2) [1]. See [37] for a thorough discussion of hybrid control schemes.

The error state vector (6) is robustly minimized with a sliding mode control law:

(7) $$V=-\mathrm{Csat}\;\left(K_{I}\int \mathrm{edt}+K_{P}e+K_{D}\dot{e}\right).$$

***K***_***I***_   ∈ ℝ^nxn^, ***K***_***P***_   ∈ ℝ^nxn^, and ***K***_***D***_   ∈ ℝ^nxn^ are the diagonal integral, proportional and derivative matrices that respectively define the slope of each sliding manifold for the six joints used in this paper. ***C*** ∈ ℝ^nxn^ is a diagonal matrix that functions as an upper bound estimate on the voltages required to overcome the torques applied on the motors. The vector saturation function, ***sat***, is used to partially linearize the control law to alleviate chatter that could occur with the signum function.

This control architecture enables all of the DOFs involved in the synergy to be controlled by a single input, *E*_*M*_ (5). Two separate methods for defining this input and driving the synergy controller are subsequently presented with varying levels of active control. For evaluation purposes, the input method can be readily switched between the two options to define *E*_*M*_ (Figure 4).

#### *EMG mapping method one: Rhythmic*

To control the sinusoidal synergy with EMG signals requires special consideration. One problem that will arise using a conventional EMG signal mapping method as in (1) is that the increase and decrease in the EMG signal (*E*) as a muscle group is contracted and relaxed will result in repetitive, counterproductive screwing and unscrewing motions. For example, if *E* (1) was used in (5), increasing *E*_*E*_ would drive the Shadow Hand through the contact stroke causing rotational motion. As *E*_*E*_ is relaxed, the Shadow Hand would follow the same Cartesian path backwards along the synergy causing rotational motion in the opposite direction.

To overcome this problem, a piecewise linear mapping is developed for the synergy so that an increase in *E*_*E*_ or *E*_*F*_ drives the synergy through the contact stroke. Afterwards, a decrease in *E*_*E*_ or *E*_*F*_ drives the synergy through the return stroke (Figure 3). *E*_*E*_ and *E*_*F*_ are first normalized such that they vary from zero when the EDC and FCR muscles are relaxed, to one at a predetermined level of contraction, respectively. In this control approach, the input *E*_*M*_ = *E*_*R*_ is defined to be

(8) $$E_{R}=\left\{\begin{array}{ll} \mathrm{\pi sat}\left(\mathrm{\omega E}\right), & \delta =0\\ 2\mathrm{\pi sgn}\left(E\right)-\mathrm{\pi sat}\left(\mathrm{\omega E}\right), & \delta =1 \end{array}\right)$$

The *sat* function in (8) is used to ensure this range of ±1 in *ωE*. The switching term, *δ*, is defined to be 0 or 1 based on whether the synergy is in the contact stroke or return stroke portion of the motion. This is determined by the measured position of a reference joint (*x*_*F*2_). Because the sine wave parameters are known prior to implementation, the maximum and minimum joint angles are known, and these correspond to the endpoints of the fingertip trajectories in Cartesian space (points A & B in Figure 3). The scaling factor *ω* determines the speed of the synergy relative to the level of muscle contraction. For example, *ω* = 3 implies that only one third of the nominal muscle contraction level is needed to complete an entire cycle.

**Figure 5:**
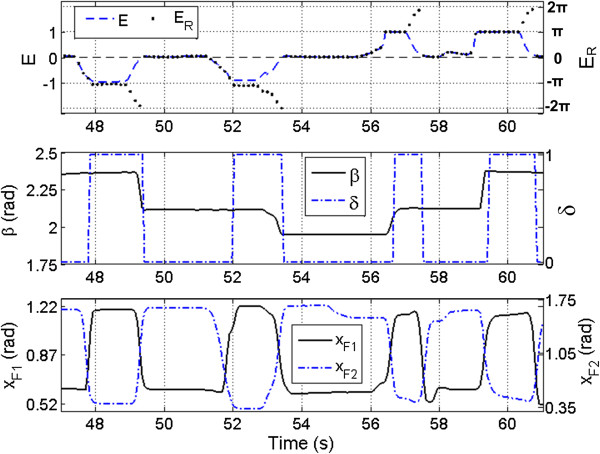
**Sinusoidal synergy controller: rhythmic method example data.** Sample data from the synergy controller performing the unscrewing and screwing task with the Rhythmic EMG mapping method. The top graph illustrates the mapping of the amplified and filtered EMG signals (*E*_*E*_ and *E*_*F*_, which comprise *E*, (1)) to the controller input *E*_*R*_. The middle graph depicts the change in cylinder position (β) as the Shadow Hand executes the contact strokes. The bottom graph shows the corresponding joint angles from the first finger required to rotate the cylinder.

Example data of the Shadow Hand performing the unscrewing task as in Figure 1(d) with the Rhythmic mapping method are shown in Figure 5. The scaled EMG input *ωE* is mapped to the synergy controller input *E*_*R*_, which ranges from -2π to 2π (Figure 5, top). This produces periodic motion of the finger joints, enabling the manipulator to rotate the cylinder in either direction. This is clear from the increase and decrease in potentiometer position (β), (Figure 5, middle). The value of δ is initially zero, causing a contraction of the EDC to drive the fingers of the Shadow Hand along the contact stroke of the synergy. When the input *E*_*R*_ = *π*, the reference joint angle *x*_*F2*_ is at its maximum. Once this condition is reached, the value of δ switches to 1, and the signal is mapped to the return stroke of the synergy as the muscle is relaxed. As *E*_*R*_ reaches zero, *x*_*F2*_ reaches its minimum, and the value of δ switches back to zero (Figure 5, middle, bottom). To produce the opposite direction of rotational motion, *E*_*F*_ is flexed and relaxed in a similar manner, driving the input *E*_*R*_ from zero to -2π. This change in sign produces the same joint and Cartesian trajectories, only mirrored in time. That is, as *E*_*F*_ is brought from zero to one, the fingertip travels through the return stroke, and as *E*_*F*_ is relaxed, the contact stroke is executed.

Through this EMG mapping method, the operator has control of the positions and velocities of the finger and thumb joints. As the positions of the fingertips are dependent upon the measured EMG signal, any point along the path of the synergy can be reached and maintained by the appropriate level of muscle contraction. Also, the speed of the synergy can be controlled by varying the amplitude and rate of contracting and relaxing the EDC and FCR muscles.

#### *EMG mapping method two: Threshold*

**Figure 6:**
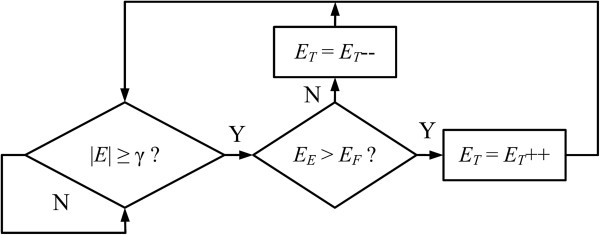
**Threshold mapping method: pseudocode logic.** Pseudocode implementation of the Threshold input mapping method. *E*_*T*_ is initialized to zero. If the magnitude of the EMG input |*E*| (1) is above a threshold γ, the input *E*_*T*_ is incremented or decremented depending upon the relative contraction levels. If *E*_*E*_ > *E*_*F*_, *E*_*T*_ is incremented to rotate the object clockwise, if *E*_*F*_ > *E*_*E*_, *E*_*T*_ is decremented to rotate the object counterclockwise.

A second method of mapping the recorded EMG signal to the synergy controller was also considered for human evaluations (Figure 4). In the Threshold mapping method, the controller input *E*_*M*_ = *E*_*T*_, and functions as a monotonically increasing or decreasing vector used to drive the sinusoids, similar to *t* in (4). *E*_*T*_ is initialized to zero when the controller is started. While the magnitude of the EMG input *E* (1) is above a deadband threshold γ, the input *E*_*T*_ is either incremented or decremented depending on the relative strengths of contraction of *E*_*E*_ and *E*_*F*_ (Figure 6). The threshold γ is empirically determined for each user, set just above the noise level for each EMG channel.

The input to the synergy controller is then given by *ωE*_*T*_, where *ω* is a free parameter which can be chosen to determine the frequency of the sinusoidal synergy. The speed of the synergy is determined ahead of time, solely by the choice of *ω*, which must be a real, nonzero value. Practically, an upper limit is placed on the choice of *ω* given the bandwidth of the physical system used, but this limit is dependent entirely upon the dynamics of the system. Above a certain value, the time constants of the motors would prevent the manipulator from fully executing the desired sinusoidal joint approximations, which could negatively affect the performance of the controller. This Threshold technique is simpler but does not have the benefit of velocity control by the operator, as is afforded by the Rhythmic method.

**Figure 7:**
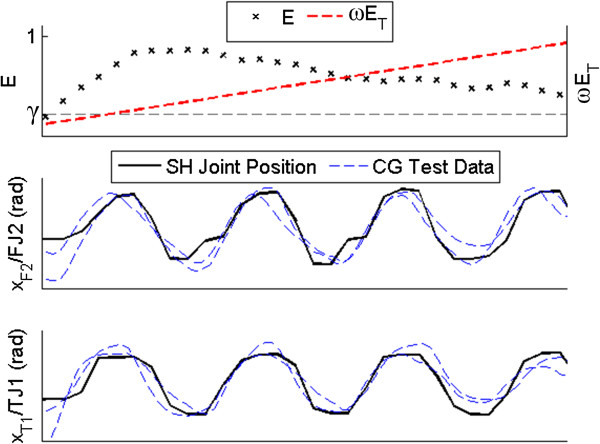
**Sinusoidal synergy controller: threshold method example data.** Comparison of Shadow Hand (SH) joint angles and two sample trials of CyberGlove (CG) data (Figure 1(a)), while the synergy controller is being operated by subject A3 using the Threshold mapping method. Once the recorded input *E* is above the deadband threshold γ, the input *ωE*_*T*_ begins to increment, driving the Shadow Hand. The time vectors and amplitudes of the CyberGlove data have been normalized for illustrative purposes. Note the similarity between the Shadow Hand and CyberGlove joint angles.

Example data of test subject A3 using the synergy controller with the Threshold mapping method are presented in Figure 7. Several joints of the Shadow Hand are compared to recorded CyberGlove joint data (Figure 1(a)) from several trials of human subjects unscrewing the potentiometer (Figure 7, middle and bottom). The joint angles of the Shadow Hand closely mimic the periodic nature of the human joint angles.

### Experimental methods: artificial hand control

The Shadow Hand synergy controller was evaluated by ten able-bodied and five limb absent human test subjects, who also compared the synergy controller to a variety of commercially available prosthetic hands. An overview of the tests performed by each subject is given in Table 2. All test subjects gave informed consent prior to experiments with the University of Akron Institutional Review Board (IRB) approval that is in accordance with the Declaration of Helsinki.

**Table 2:** Summary of participants and tests performed

**Subject(s)**	**Limb absence**	**Acquired/congenital**	**Test conditions**		
			**Human hand (H)**	**Shadow hand mapping (R, T)**^**#**^	Prostheses used*
*1-10*	N/A	N/A	H	R	MC [1(e)], ILU [1(f)]
*A1*	Right	Acquired	H	R, T	SHS [1(g)]
*A2*	Bilateral	Congenital	N/A	R, T	VPS [1(h)]
*A3*	Left	Acquired	H	R, T	VPS [1(i)]
*A4*	Left	Acquired	H	R, T	ETD [1(j)], IL [1(k)], BP [1(l)]
*A5*	Right	Congenital	H	R, T	N/A

^#^Abbreviations R and T refer to the Rhythmic and Threshold EMG Mappings, respectively.

^*^Characters in brackets refer to photos of the prostheses presented in Figure 1.

To measure the angle of object rotation (β), a 35 mm diameter cylinder was mounted to a potentiometer (Figure 1(d)). The subjects were asked to fully rotate the cylinder 6 rad as quickly as possible, similar to the previously described task in the human hand motion analysis (Figure 1(a), Appendix A).

#### *Test participants: able-bodied and limb absent test subjects*

Two separate subject groups participated in the current study, summarized in Table 2. The ten able-bodied test subjects (1-10) performed the task with the Motion Control Hand (Figure 1(e)), the i-Limb Ultra (Figure 1(f)), and the Shadow Hand with the Rhythmic EMG mapping method.

Five limb absent test subjects with various natures of hand absence completed the unscrewing task using the Shadow Hand with both EMG mapping methods and their own prostheses that they use in their daily lives (Table 2). The first limb absent test subject (A1) has a right transradial amputation. He has used a powered prosthesis for nearly a decade. His current prosthetic hand for daily use is the SensorHand Speed [38] (Figure 1(g)). The second test subject (A2) in this study has a bilateral congenital hand absence. She has been using two Otto Bock MyoHand VariPlus Speeds for several years (Figure 1(h)). The third subject (A3) has a left transradial disarticulation and has been using a powered prosthesis for two and a half years. He also uses a MyoHand VariPlus Speed (Figure 1(i)). The fourth subject (A4) also has a left transradial disarticulation. He uses various prostheses in his activities of daily living: a Motion Control ETD Hook (Figure 1(j)), an i-Limb (Figure 1(k)) [3], and a body powered Grip Prehensor prosthetic hand (TRS Inc., Boulder, CO, USA) (Figure 1(l)). This test subject also has had an amputation of his first, middle, and ring fingers on his right hand at the PIP joints due to the nature of his accident. The fifth limb absent subject (A5) has a congenital absence of her right hand and had never before used a myoelectric prosthesis in her activities of daily life.

#### *Experimental procedure: able-bodied test subjects*

The ten able-bodied participants (Subjects 1-10) performed the rotational task with their natural hand, Shadow Hand, Motion Control Hand, and i-Limb Ultra systems (Table 2). For comparison purposes, each subject was initially asked to fully rotate the cylinder with their own hand 10 times, with the additional instruction to complete the task as quickly as possible.

After this procedure was completed, participants then performed the task with the Motion Control Hand (Figure 1(e)), the i-Limb Ultra (Figure 1(f)), and the Shadow Hand synergy controller (Figure 4) with the Rhythmic EMG mapping method (8).

Two EMG preamplifiers were placed atop the EDC and FCR muscles of the subjects’ forearms prior to testing. EMG signals were rectified, filtered and amplified using Myolab II (Motion Control, Inc.). A BNC-2090A DAQ Board (National Instruments, Inc.) interfaced with MATLAB/Simulink via a PCI-6229 card was used to sample EMG data at 1 kHz with the real time Windows target kernel. These filtered EMG signals were used to control the Motion Control Hand and i-Limb Ultra.

Half of the able-bodied test subjects began with the Motion Control Hand (Group 1), and the other half performed the task with the Shadow Hand first (Group 2). This was done since the participants were unfamiliar with EMG control, to reduce the impact of learning effects of the test subjects, on average, from influencing the performance with a particular system. All trials with the i-Limb Ultra were performed subsequently. A practice time of five minutes with each system was given prior to recorded trials. Each subject performed the task 10 times with each artificial hand and the time to complete the task was tabulated.

A single factor ANOVA test was performed between the controllers to determine if a statistically significant improvement was offered by the synergy controller with respect to the amount of time required to complete the task with the other two systems. Three single factor ANOVA tests were also performed to examine the performance differences between able-bodied Groups 1 and 2 for each artificial system. These tests determined if there were any learning effects in the performance of each system as the subjects became more familiar with EMG control. They also indicated whether or not a substantial improvement was enabled by the additional dexterity of the i-Limb with respect to the one DOF Motion Control Hand.

#### *Experimental procedure: limb absent test subjects*

In the case of the limb absent subjects A1-A4, their own prostheses were used to perform the rotational task. This subject group evaluated both EMG mapping methods for the Shadow Hand (Rhythmic and Threshold). They also performed the task with their natural hands (except subject A2 who has a bilateral limb absence). Subject A5 only used the Shadow Hand with both mapping methods since she does not own a prosthesis and had never used one previously. Every participant performed each test condition 10 times (Table 2).

After data collection, a single factor ANOVA test was performed on the individual completion times for each method and subject. This was used to quantify the performance of each individual with respect to every control method due to the anatomical differences among test subjects. These tests were further analyzed using the multcompare function in MATLAB, which uses the results from a single factor ANOVA test to determine the level of significance between systems for each individual subject.

Additionally, a two factor ANOVA test was performed with the artificial hand data (consisting of time trials from the prosthetic and Shadow Hand systems) from subjects A1-A4. This test examined the performance of each control method (factor A) across the group, as well as to whether or not the operator (factor B) played a significant role in the performance. The ETD Hook was the only prosthesis considered in this test for Subject A4, to limit the comparison to single DOF myoelectric prostheses used. To have a balanced test, subject A5 was not considered in this test, as she lacks an integrated prosthesis.

The Rhythmic and Threshold mapping methods for the Shadow Hand were also evaluated subjectively by the limb absent participants. Every subject rated how similar each EMG mapping method felt in comparison to the human hand while completing the task on a scale of 1-10. A nonparametric Mann-Whitney *U*-Test was performed on their responses to evaluate the significance of the results.

When controlling the Shadow Hand, the EMG preamplifiers from the MyoLab II were placed atop the same recording sites that the test subjects A1-A4 use with their own prostheses. Test subject A5 was found to have excellent EMG signals for control in the distal portion of her residual limb.

## Results

### Able-bodied test subject results

#### *Average task completion time*

**Figure 8:**
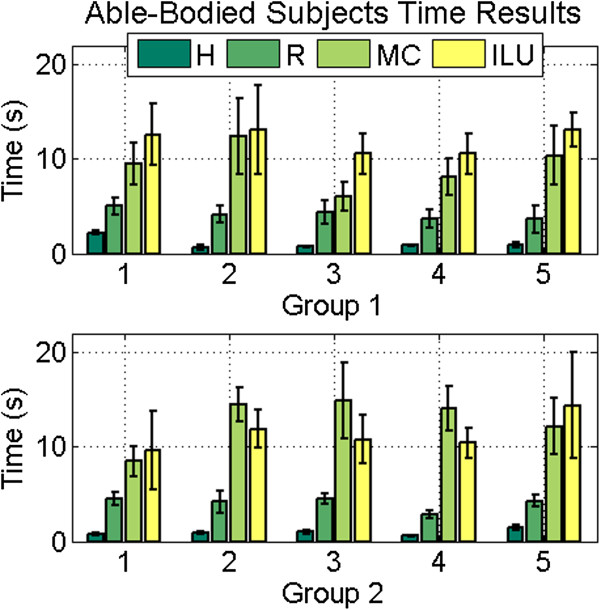
**Able-bodied subjects: time results.** Time results for able-bodied subjects, by group. All subjects completed the task faster with the synergy controller using the Rhythmic mapping method for the Shadow Hand (R) than the Motion Control (MC) Hand or i-Limb (ILU) Hand. All subjects performed the task fastest with the human hand (H).

The average task completion time was calculated for each of the four methods (human hand, Motion Control Hand, i-Limb Ultra, and the Shadow Hand with the Rhythmic EMG mapping method), Figure 8. As expected, subjects using their own hands completed the task in the fastest time, with an average of 1.54 s across all subjects. The task was completed in 11.04 s on average while using the Motion Control Hand, and in 11.69 s with the i-Limb Ultra. The average completion time for the Shadow Hand sinusoidal synergy controller was 4.10s across all subjects. This represents a 177% improvement in completion time with the Shadow Hand.

#### *Statistical analysis of task completion time*

The completion time of every trial for all ten able-bodied subjects was used for the ANOVA test, resulting in 100 data points per system (10 trials from each subject per system). As expected, there was a statistical difference between each of the robotic systems and the human hand, with a high level of confidence (p < 0.01). The Shadow Hand synergy controller offered a significant improvement over both the Motion Control Hand and the i-Limb Ultra (p < 0.01). There was no significant difference between the Motion Control Hand and the i-Limb Ultra (p > 0.05). Three additional ANOVA tests evaluated the mean performance of both groups for each artificial system. All three tests proved non-significant (p > 0.05), indicating that learning effects did not significantly affect the performance between the Motion Control Hand and the Shadow Hand between the two test groups. It also indicates that the additional dexterity of the i-Limb Ultra provided no benefit to complete this particular task more quickly than the one DOF Motion Control Hand.

### Limb absent test subject results

#### *Average task completion time*

**Figure 9:**
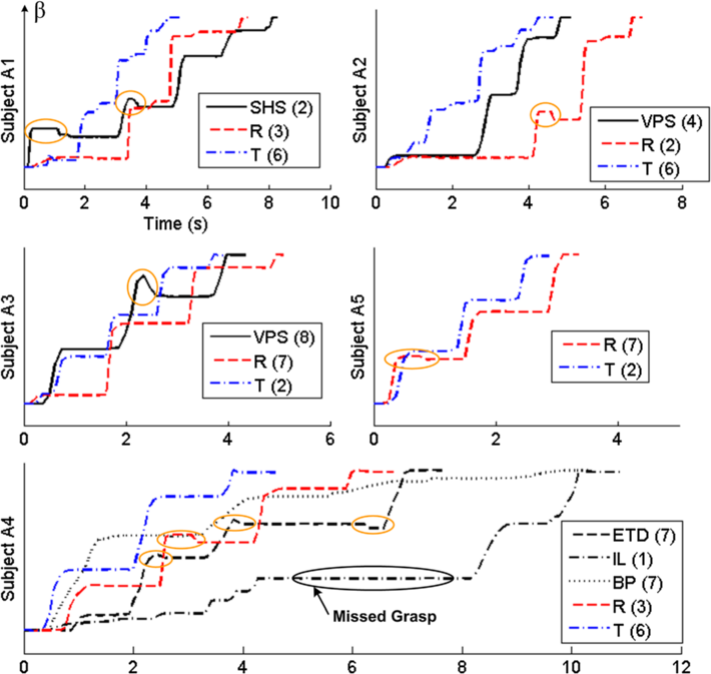
**Limb absent subjects: example data.** The slowest individual trials for each limb absent subject for each robotic system used to perform the task. The abscissa is time in seconds, while the ordinate is the normalized cylinder position (β). Black lines represent the subjects’ own prostheses (see Figure 1 caption for abbreviation definitions), while the red and blue dashed lines represent the Shadow Hand using the Rhythmic (R) and Threshold (T) input mappings, respectively. Numbers next to each legend entry denote the trial number (out of 10). Cases where the object was accidentally rotated backwards are circled in orange. The Threshold method performed the task the fastest in this dataset.

Results of the slowest individual trials for each robotic system used by subjects A1-A5 are shown in Figure 9. In all of these cases the synergy controller with the Threshold input mapping produced the fastest times. The synergy controller also reduced accidental backward rotation of the cylinder during the task. It was observed while using their own prostheses that an errant arm motion or EMG input would cause a subject to counter-rotate the object or miss the grasp entirely, as occurred during subject A4’s first attempt while using his i-Limb (Figure 9). While this could happen because of a poorly-timed muscle contraction with the Rhythmic method, it never occurred while using the Threshold method.

**Figure 10:**
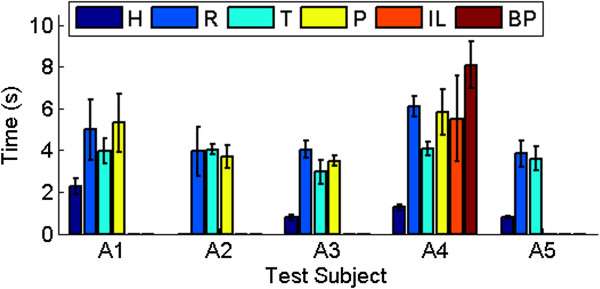
**Limb absent subjects: time results.** Task completion time results for individual limb absent subjects, using their own hand (H), the synergy controller with the Rhythmic (R) and Threshold (T) input mappings, and up to three of their own prostheses (P), as described in Figure 1 and Table 2. For test subject A4, this denotes the ETD Hook (Figure 1(j)). This subject also performed the unscrewing task with his i-Limb (IL) and body powered Grip Prehensor (BP).

The average times to complete the task using the Shadow Hand, the subjects’ natural hands, and their respective prostheses were calculated for all subjects (Figure 10). As expected, the subjects completed the task in the shortest time while using his or her natural hand (1.28 s average). This does not include test subject A2 who has a bilateral congenital limb absence.

With respect to the artificial hands, the Shadow Hand with the Threshold mapping method produced the fastest average time to complete the task; faster on average than with their own prostheses (3.73 s versus 6.06 s), representing a 62% improvement in completion time. The Rhythmic mapping method was the second fastest with an average completion time of 4.58 s, a 32% improvement over the subjects’ own prostheses. Overall, the newly introduced Threshold method was faster than the Rhythmic EMG mapping method by 0.85 s, or 19%.

It should be noted that the average completion time of 6.06 s with the limb absent subjects’ own prostheses includes data from subject A4’s body powered prosthesis (BP). Disregarding these body powered data results in an average completion time of 5.04 s while the limb absent subjects were using their own prostheses, with the proposed synergy controller still producing a 9% and 26% improvement using the Rhythmic and Threshold mappings, respectively.

Test subject A1 was able to complete the task faster with the synergy controller than with his own prosthesis by 0.32 s and 1.32 s using the Rhythmic and Threshold methods, respectively (Figure 10). Test subject A2 completed the task in 3.68 s with her own prosthesis; with the synergy controller, she had average completion times of 4.03 s and 3.94 s using the Threshold and Rhythmic mapping methods, respectively. Test subject A3 was faster with the Threshold mapping method than with his own prosthesis by 0.53 s, but was slower with the Rhythmic method by 0.54 s. Test subject A4 was also faster with the Threshold method (4.07 s) than with each of his own prostheses by 2.40 s on average. The slowest average time for subject A4 was seen while using the body powered prosthesis (8.09 s), with the i-Limb hand producing the second fastest time on average: 5.51 s (Figure 10).

Subject A5 proved to be proficient with both synergy controller EMG mapping methods, completing the task in 3.85 s and 3.61 s with the Rhythmic and Threshold mapping methods, respectively.

#### *Statistical analysis of task completion time*

**Figure 11:**
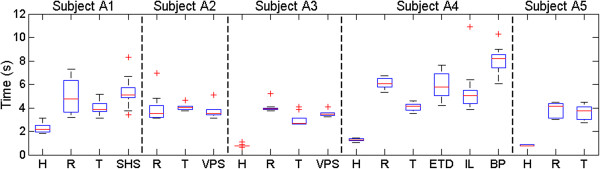
**Limb absent test subjects: statistical analysis.** Box and whisker plots of the limb absent participants’ time results for each method of control: human hand (H), synergy controller with the Rhythmic input mapping (R), and Threshold input mapping (T), and prosthesis(es). Refer to the caption of Figure 1 for abbreviations of the commercial prostheses tested by each subject. The human hand is significantly fastest in all applicable cases. The Shadow Hand Synergy Controller with the Threshold mapping method was the significantly fastest artificial method for subjects A1, A3, and A4. Subjects A2 and A5 showed no significant difference between the robotic systems they tested.

Unsurprisingly, the single factor ANOVA tests performed on the individual subjects indicated a statistically significant difference in completion times (p < 0.001). This is because the results from the human hand are much faster than all the artificial hands in all relevant cases (except for subject A2 who lacks a natural hand), Figure 11.

The two factor ANOVA test on the artificial hand data returned significant results from both the controller/artificial hand (factor A) and test subjects (factor B): p_A_ < 0.001 and p_B_ < 0.001, as well as a highly significant interaction between the two factors (p_A/B_ < 0.001).

With respect to the artificial hands, Subjects A1, A3, and A4 were significantly fastest using the Threshold EMG mapping method (p < 0.05). Subject A2 showed no significant difference between any of the robotic systems (p > 0.05) (Figure 11). Test subject A5 showed no statistical difference between the two EMG mapping methods for the Shadow Hand.

For Subjects A1 and A4, no significant difference existed between their prostheses and the synergy controller with the Rhythmic EMG mapping method. Subject A3 was statistically slower using the Rhythmic method than with his own prosthesis.

Test subject A4 was significantly slower while using his body powered prosthesis (Figure 1(l)) but no significant difference was seen between his two myoelectric prostheses (Figure 1(j,k)) and the synergy controller with the Rhythmic EMG mapping method (Figure 11).

In all, the Shadow Hand synergy controller with the Threshold mapping method was significantly faster than the commercially available prostheses for subjects A1, A3 and A4; it was not significantly slower for subject A2.

#### *Subjective results*

**Figure 12:**
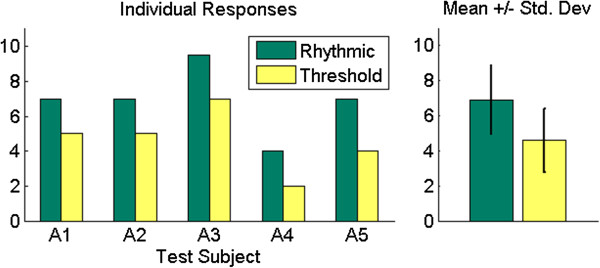
**Limb absent subjects: subjective results.** Individual results of subjective evaluations (left), and average responses (right). The Rhythmic EMG mapping method was rated to be more similar to control of the human hand by all test subjects.

Each limb absent person also subjectively evaluated how similar the Rhythmic and Threshold mapping methods were to control of the human hand. In each case, the Rhythmic method was rated higher (Figure 12). On average, the participants rated the Rhythmic mapping method as 6.90 ± 1.95, and the Threshold mapping as 4.60 ± 1.82. The results of the *U*-test returned a p-value of p = 0.135, which is not statistically significant.

## Discussion

### Sinusoidal synergy controller

There are several observations about the proposed sinusoidal synergy controller that merit mention. First, the approximation of the human finger and thumb joint motions by sinusoids provides numerous advantages for control of an artificial hand. Because the human finger joint trajectories are periodic and share the same frequency, a single set of sinusoidal inputs can be used in the path planning of the robot to perform this task with high accuracy. Also, by adjusting the position offsets of the sine waves, the manipulator can be made to unscrew different diameter objects [18]. Additionally, this synergy allows the controller to switch from an unscrewing motion to a screwing motion by decrementing the time vector in (4), as opposed to incrementing it [18]. This reverses the motions of all the joints simultaneously, and induces the opposite direction of angular rotation of the object.

In addition to completing the task more quickly than commercial prostheses on average, the developed synergy controller has several other distinct advantages. For example, the proposed controller produces more anthropomorphic motions, with proportional control of the position and velocity of the joints along the path of the synergy (in the case of the Rhythmic EMG mapping method). Also, the synergy controller presented herein has the additional benefit of being functional in a tight working environment, which is not possible for unscrewing and screwing motions with commercially available prosthetic hands. Finally, less physical effort is required with the proposed synergy controller since no additional motion of the shoulder and elbow is required to rotate the object as is necessary with commercially available prosthetic hands.

**Figure 13:**
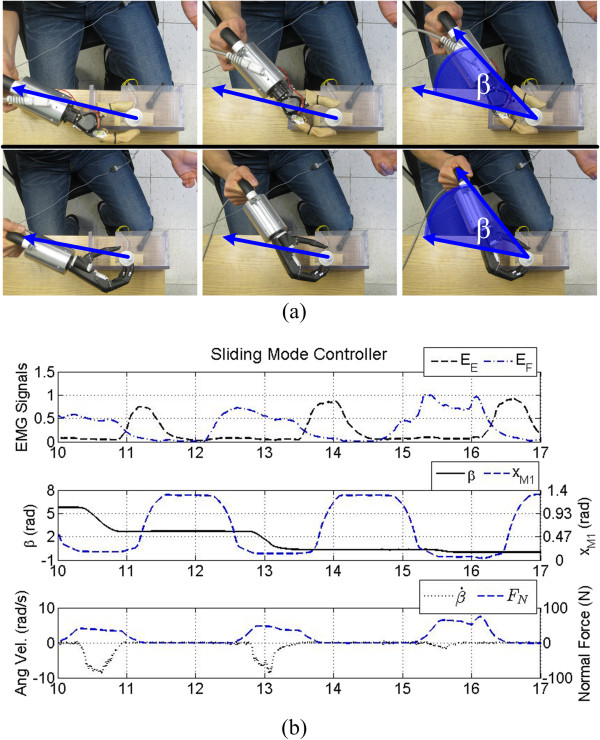
**Prosthetic hand control. (a)** Photo sequence of an able-bodied test subject performing the unscrewing task with the Motion Control Hand (top), and i-Limb Ultra (bottom). The prostheses must be manually rotated using elbow and shoulder motions to produce rotation of the object once in grasp. The angular position of the object is denoted as β. In essence, prosthetic hands function similar to a crescent wrench during this task. **(b)** The Motion Control hand under sliding mode control. A contraction of the EDC (*E*_*E*_) causes the hand to open, while a contraction of the FCR (*E*_*F*_) causes the hand to close. The prosthesis must be manually rotated as in (a) to rotate the grasped object.

In this sense, current prosthetic hands function similar to a crescent wrench to unscrew objects which limits their viability in tight workspaces (Figure 13(a)). To demonstrate this, sample screwing data from the Motion Control Hand is shown in Figure 13(b). Here, the difference in the measured EMG signals (*E*_*E*_ and *E*_*F*_) from the forearm is used to open or close the hand by alternating the relative contraction strength of each muscle (Figure 13(b), top). When the Motion Control Hand has the potentiometer in grasp, F_N_ increases (Figure 13(b), bottom) and the prosthesis must be rotated using shoulder and elbow movements (Figure 13(a), top) to induce a change in the potentiometer angle, β (Figure 13(b), middle). Repeating this process continues the unscrewing motion.

Looking forward, the synergy control concept in Figure 1 can also be expanded and adapted to other tasks by altering the sine wave parameters, or even choosing other periodic functions to control different kinds of cyclical tasks via a single control input. The synergy control method outlined in this paper could be used with any input source, not just EMG. For example, electroencephalogram (EEG) signals could be used by other populations of disabled people like stroke victims and quadriplegics to have dexterous control of an anthropomorphic manipulator. The Threshold mapping method would be particularly useful for EEG control, and is currently being explored for this purpose.

Finally, it is worth discussing the performance of the proposed sinusoidal synergy technique in less structured, real world situations. As an example, the position of the object was locked in the current study. This is similar to many real world situations, such as working on an automobile or manipulating dials on a piece of equipment. However this is certainly not always the case, for example, when the object to be unscrewed is grasped in the other hand. In this kind of bimanual task, the operator would also need to maintain the correct position of the artificial hand relative to the object in order to complete the task. As with commercially available prosthetic hands, the performance of the sinusoidal synergy technique is affected by the positioning of the object within the workspace of the fingers. An advantage of the proposed synergy controller is that after the correct positioning has been achieved, the hand can be held in place while the screwing/unscrewing motions are produced entirely by the fingers. This is in contrast to performing the same task with currently available prostheses, which must be repositioned, closed, and manually rotated with arm and shoulder movements to achieve each cycle of the rotational motion (Figure 13).

Despite these inherent advantages with the synergy controller, it is likely that tactile feedback could be a useful addition to improve the robustness required for implementation in tasks of daily living [39]. It is hypothesized that tactile fingertip force feedback could also be used to further improve the time to complete the unscrewing task, since the human hand, which offers rich tactile information, was always statistically fastest. The addition of tactile feedback may also help to further reduce the sensitivity of the proposed technique to uncertainties in object location and geometric eccentricities.

### Able-bodied test subjects

Results from the timed tests show significantly faster completion times with the synergy controller using the Rhythmic mapping method (4.10 s average) than with the Motion Control Hand (11.04 s average) and i-Limb (11.69 s average). This represents an improvement of 177% over the prosthetic systems on average. Additionally, the range of mean times between subjects and individual standard deviations are lower with the synergy controller. Together, these findings suggest that the developed controller provides a sufficiently intuitive human-machine interface with low required training time to become proficient.

The ANOVA of the task completion time for the able-bodied subjects demonstrated that there is no statistically significant improvement offered by the increased dexterity of the i-Limb (with one active DOF per digit, versus one DOF total for the Motion Control Hand) for this particular task. There was, however, a significant difference in time to complete the task between the i-Limb Ultra and the Shadow Hand (which had two active DOFs per digit). This demonstrates the need for more dexterous prosthetic hands, and illustrates one way this additional functionality could be used. This information is useful for prosthetic hand manufacturers to consider for future designs of more dexterous prostheses.

The inclusion of tendon force feedback for the synergy controller in this paper is an important improvement over prior work which only had position feedback [34]. By increasing the compliance of the closed loop system with the hybrid control scheme in this paper, a faster synergy time was enabled relative to results from the able-bodied participants who used a version of the synergy controller which had no tendon force feedback. Without feedback, an average completion time of 5.17 s was achieved with the same system [34]. The addition of tendon force feedback produced an average time of 4.10s, representing a 21% improvement over earlier efforts. The increased compliance offered by the tendon force feedback also makes the system less susceptible to improper placement of the object. If, for example, the object is closer to the hand than intended, the resulting increased tendon force will autonomously extend the finger and thumb joints further and allow the digits to complete the unscrewing motion more easily. This also serves to regulate the applied force to the rotating object, reducing the possibility of squeezing the object too tightly. As mentioned earlier, the addition of tactile feedback is also being investigated to further improve the robustness and performance of the sinusoidal synergy technique.

### Limb absent test subjects

The limb absent subjects were noticeably faster using their own prostheses than the able-bodied participants who used the Motion Control Hand and i-Limb Ultra. As a group, the limb absent group had an average completion time of 6.06 s while the average for the able-bodied subjects was 11.37 s (Figures 8 and 10). This was expected, as the prostheses of those test subjects with a hand absence are well integrated into their residual limbs. Additionally, the able-bodied subjects had little to no prior experience with EMG control of a prosthesis. Despite this, the average completion times are roughly comparable between the limb absent and able-bodied subjects while using the Rhythmic EMG mapping method for the Shadow Hand synergy controller: 4.44 s versus 4.58 s, respectively. This is encouraging because it shows that even though the limb-absent subjects (A1-A4) have much more experience with EMG control, the able-bodied subjects and subject A5 were able to control the Shadow Hand approximately as well as subjects A1- A4, who have integrated prostheses. The low training time required for proficiency is also illustrated by subject A5, who achieved fast task completion times despite never previously using a prosthesis in her daily activities.

The limb-absent subjects were asked to subjectively evaluate how similar the Rhythmic and Threshold EMG mapping methods are to control of the human hand. All subjects evaluated the Rhythmic method higher (Figure 12). When asked to explain their numerical evaluations, the common response was that the Threshold method felt more artificial than the Rhythmic method because a simple maintained contraction of a muscle group caused motion of the Shadow Hand with the Threshold method. Overall, the participants felt that the Rhythmic mapping method allowed for a more natural feeling way to control the Shadow Hand while rotating the cylinder. However, test subject A4 did mention that the Threshold method was easier for him to use, which is due to his significantly faster performance with the Threshold method (Figure 11).

Even though the Rhythmic method was subjectively rated higher than the Threshold method (though not significantly due to the sample size), results from the timed task show that both methods are effective. This is of particular interest because the results of the two-way ANOVA show that there is a significant interaction between the test subject and the EMG mapping method. Because subject A4 had a slower completion time with the synergy controller using the Rhythmic EMG mapping method, he would probably benefit more from use of the Threshold method for his daily tasks. This is because the Threshold method is simpler to operate and produced the fastest task completion time on average among all limb absent test subjects (Figure 11). Other test subjects who had no significant difference between the two mapping methods (A2 and A5) might opt to use the Rhythmic method because of the increased ability to control both the position and velocity of the hand along the path of the synergy (8).

All limb absent subjects were significantly faster with the Threshold method than they were with their own prostheses except subject A2, who showed no significant difference. Several other factors should also be mentioned that were not measured. First, the nature of completing this task with a conventional prosthesis requires a large workspace to manually unscrew or screw the grasped object (Figure 13). This is unsuitable in small working environments, and limits the functionality of the prosthesis under these conditions. The proposed synergy controller, however, requires a workspace only large enough to accommodate the motions of the fingers (Figure 1(d)). Once the object is appropriately positioned between the fingers, no more manual adjustment is needed to rotate the object. This also reduces the tendency to inadvertently rotate the cylinder backwards as occurred with the commercially available prostheses (Figure 9). In addition to the benefit of a reduced workspace, the synergy controller also requires much less physical energy from the operator. This was most exemplified in subject A4, who was physically tired after completing testing with his prostheses, especially with his body-powered prosthetic hand. The sinusoidal synergy controller reduces the required physical efforts since no elbow and shoulder motions are necessary after positioning the hand.

## Conclusions

This paper has presented results from a timed rotational task using natural and prosthetic hands, evaluated by participants with and without a limb absence. The results demonstrate the need for more dexterous prosthetic hands. Thus, a clinically viable solution to this problem was presented for a dexterous artificial hand. The newly developed sinusoidal synergy controller was compared to multiple commercially available prosthetic hands by five subjects with a hand absence and ten able-bodied test participants to rotate an object as quickly as possible.

Test subjects using the sinusoidal synergy controller completed the task more quickly than they did with the commercially available prostheses. The synergy controller provides several other distinct advantages, including a reduced workspace and less physical effort required to operate. Moreover, the process used to derive this synergy (summarized in Figure 1) can be adapted for different tasks or input signals with little modification. The current work has made several significant improvements over prior work [18,34], including the implementation of tendon force feedback to improve the performance, minimization of the number of joints required to complete the task, and an additional EMG synergy mapping method. The newly developed Threshold EMG mapping method improved task completion time by 19% with the limb absent subjects. New experimental results presented herein have demonstrated the efficacy of these improvements.

Future work for this research is to expand this sinusoidal synergy concept to function for any arbitrary orientation of the hand with respect to the grasped object [40,41]. A top level controller, currently under development, will include the ability for the operator to switch between other synergistic motions and grasp patterns. This will allow the user to perform a wide variety of actions by utilizing finger joint motion synergies inherent in natural hand control.

## Appendix A. Initial human hand study

Prior to the artificial hand comparison study, an initial human hand motion study was performed (Figure 1(a)). The results from these experiments were used to derive the sinusoidal joint motion approximations used in the Shadow Hand synergy controller. The following outlines the experimental procedures and results of this initial study.

### Equipment - CyberGlove II

**Figure 14:**
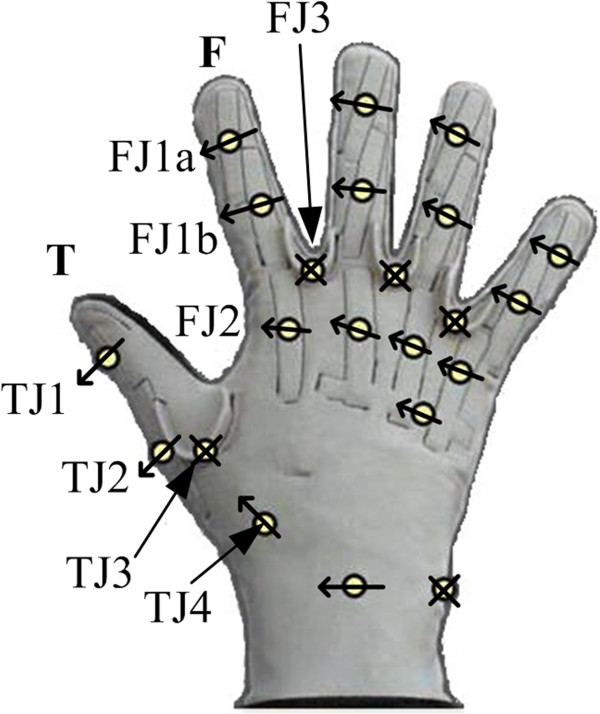
**CyberGlove II sensor placement.** The CyberGlove II has 22 sensors to measure the motions of human hands.

The hand motion profiles of ten human test subjects were recorded using the ver. 2.2 CyberGlove II (Figure 14), (Immersion Corporation, San Jose, CA). The glove converts finger and thumb joint angles into digital joint angle data in real time, and has been used in previous studies to record motions of the human hand for robotic applications [18,42]. Each of the fingers of the glove contains four sensors, two of which record joint angle data for the distal interphalangeal and PIP joints. The remaining two measure extension/flexion and abduction/adduction of the MCP joint. There are four sensors in the thumb to measure the motions of the IP, CMC, and MCP joints.

### Experimental methods: CyberGlove

Ten able-bodied human test subjects gave informed consent prior to experiments with University of Akron IRB approval that is in accordance with the Declaration of Helsinki. Each test subject was first fitted with a brace to immobilize the wrist during testing. Prior to data acquisition, the forearm of each subject was secured in place and a bottle was placed at a measured orientation angle θ = π/2 rad relative to the wrist. Here, the orientation angle is defined as the angle between the axis of rotation of the bottle cap and the longitudinal axis of the forearm that runs from the elbow to the hand (Figure 1(a)). A standard 500 mL plastic water bottle with a cap diameter of 30 mm was used during this experiment. The participants were instructed to unscrew the cap completely using only the first finger and thumb while both the wrist and bottle were secured in place, during which time joint angle data for these digits were recorded at 40 Hz. The subjects were given no time constraints to complete the task, but instead were asked to unscrew the cap in the most natural manner given the applied physical constraints. The wrist brace served to prevent movement of the wrist from imparting any motion to the cap while the finger joint angles were being recorded because simultaneously powered wrist actuation is not a capability available to most prosthetic hands. Additionally, this isolated the effects of the finger and thumb motions during the task. This procedure was repeated five times for each test subject.

After data collection, the hand motion profiles of the individual trials were observed with a human hand skeletal structure in Simulink using the 3D Animation toolbox (Figure 1(a)). A principal component analysis (PCA) was performed on the joint angle data to determine the impact of each joint during the task. PCA is commonly used as a data reduction method to eliminate redundant variables in high dimension problems. As will be subsequently demonstrated, the periodic nature of the unscrewing joint angle motions enabled the accurate approximation of the human joint angle profiles by a set of sinusoidal trajectories.

#### Human hand joint space data

The joint angle data from the CyberGlove experiments were first filtered and normalized with respect to time so that each subject performed the same number of cycles per second of normalized time. This was done because some of the subjects performed the task more quickly than others.

**Figure 15:**
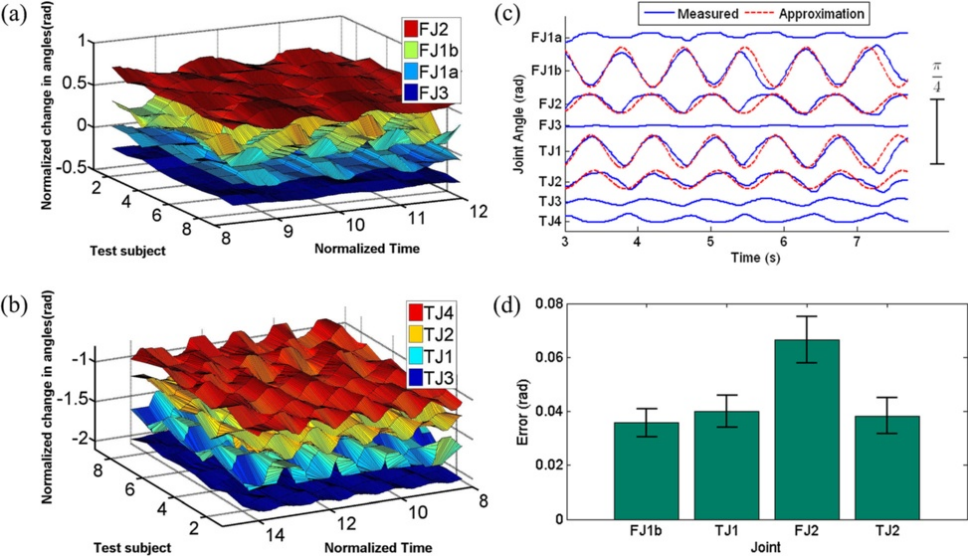
**Joint space contour plots. (a)** Contour plots of the recorded joint angle data of the first finger and **(b)** thumb. Notice the similarities in frequency and amplitude among the ten subjects as they unscrewed the bottle cap. **(c)** All recorded joint angle data for a single trial from one test subject. The joint amplitudes remain relatively constant throughout completion of the task, and the frequencies of all joints remain constant with respect to one another. However, there are joint angle offsets between the joints of the hand. Sinusoids are superimposed over the four joints used to recreate this task with the robotic system to further demonstrate the periodic nature of the joint motions. **(d)** A joint space error analysis was performed between the sinusoidal approximations and the recorded human joint angles. The low errors and standard deviations suggest that the developed sinusoids reproduce the observed human motions with sufficient accuracy to complete the task with the robotic system.

Observation of the data revealed two tendencies: first, that the individual finger joints exhibited a periodic motion which can be closely approximated by a sinusoid. Second, the frequency of this periodic motion remained relatively constant for all joints throughout the duration of each trial. However, there are phase differences between the respective joints (Figure 15).

Contour plots from a single trial for each joint from all of the ten test subjects were constructed with respect to normalized time (Figure 15(a,b)). These tendencies are further illustrated in Figure 15(c), which shows all recorded joint angles during a single unscrewing trial for one test subject. Four of these joint angles were later approximated by sinusoids for implementation on the robotic system (Figure 15(c)), as previously described.

#### Principal component analysis results

**Figure 16:**
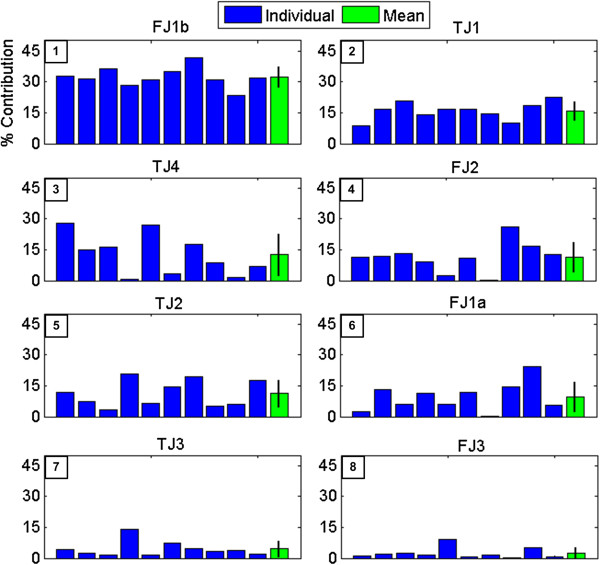
**Principal component analysis results.** Results of the PCA. The percentage contribution of every joint is shown for each individual subject (1-10), as well as the average across all subjects. The number in the upper left corner of each graph denotes the rank of contribution for each joint.

Results of the PCA show that the first principal component (PC) for each test subject accounts for 72.6% of the variance on average. The scalar coefficients of the first PC for each test subject were converted to percentages to determine the contribution of each joint variable to that PC (Figure 16) for both the individual subjects and the group on average. This analysis was limited to the first PC only due to the high amount of variance attributed solely to the first PC, a result of the highly constrained nature of the task. Figure 16 ranks the most impactful joints (those contributing most to the variance) from one to eight according to the PCA, plotted in descending order.

#### Sinusoidal joint angle approximations

The aforementioned PCA was used to identify joints that contributed strongly to completion of the task and to identify which joints were not required. Additionally, the PCA was used to establish the relative amplitudes of each joint with respect to the remaining joints (A_*k*_), while the joint space data were used to establish the required phase offsets *ϕ*_*k*_. Finally, a single set of sinusoidal parameters were developed and used with the Shadow Hand for all test subjects (Table 1).

A joint space error analysis was performed using the average absolute relative error between the developed sinusoidal approximations and the recorded joint angles of the human subjects. The results of this analysis show that the sinusoidal approximations closely reproduce the human joint motions (Figure 15(d)). This joint space error analysis demonstrates that the developed sinusoids recreate the motion in the joint space with sufficient accuracy to complete the task with the robotic system.

One of the major differences in this paper from prior work [18] is the goal to use as few DOFs of the Shadow Hand as possible, which will facilitate the application of this technique to prosthetic hands. To that end, it is clear from Figure 16 that joints FJ1b and FJ2 have the largest impact with the first finger. Accordingly, a two DOF model was used for the first finger (*x*_*F1*_ and *x*_*F2*_) since the abduction motions of the human test subjects were minimal.

TJ1, TJ4, and TJ2 have the most influence on the motions of the thumbs of the test subjects. As one of the goals of the current work is to minimize the number of joints required to complete this task, two actuated joints of the thumb were used (*x*_*T1*_ and *x*_*T2*_) while the other two joints of the thumb (*x*_*T3*_ and *x*_*T4*_) were given constant position offsets to appropriately position the thumb. Joint *x*_*T2*_ was chosen over *x*_*T4*_ for several reasons. Both TJ2 and TJ4 have nearly equal contributions to the task during human testing (Figure 16) but the inter-subject variation for TJ4 is higher, most likely due to greater anatomical variability with this joint of the human thumb [43]. This variability led to some subjects using TJ2 more than TJ4 during the task. Additionally, newer multi-DOF prostheses (such as the i-Limb and bebionic hands [36]) commonly implement a passive thumb circumduction joint with one actuated MCP flexion joint. Thus, the use of constant angles for *x*_*T3*_ and *x*_*T4*_ are representative of the existing passive circumduction joints that are already implemented in commercially available hands. However, *x*_*T1*_ (and *x*_*F1*_) represent a new feature that is not currently available in any prosthetic hand that could be evaluated by manufacturers as a potential addition to future designs.

#### CyberGlove to shadow hand mapping

After establishment of the sinusoidal joint approximations (*h*_1_(*t*)–*h*_*m*_(*t*)), these functions were then mapped to the robotic system ($x_{D_{1}}\left(t\right)–x_{D_{n}}\left(t\right)$) to form the desired joint trajectories for the Shadow Hand (Figure 1(a), Table 1). To do this, joints *x*_*T1*_, *x*_*T2*_, *x*_*T3*_, *x*_*T4*_ were mapped from joints TJ1, TJ2, TJ3, and TJ4 of the human hand, respectively. The two DOFs of the first finger used in this paper are the PIP and MCP joints, *x*_*F1*_ and *x*_*F2*_, which were mapped to the Shadow Hand from joints FJ1b and FJ2 of the human hand, respectively [18].

## Abbreviations

EMG: Electromyogram; DOF: Degree of freedom; PIP: Proximal interphalangeal; EDC: Extensor digitorum communis; FCR: Flexor carpi radialis; CMC: Carpometacarpal; MCP: Metacarpophalangeal; IP: Interphalangeal; PCA: Principal component analysis; PC: Principal component; EEG: Electroencephalogram; MC: Motion control hand; ILU: i-Limb Ultra; SH: Shadow hand; SHS: Sensor hand speed; CG: CyberGlove; VPS: MyoHand VariPlus speed; IL: i-Limb; BP: Body Powered Grip Prehensor; ETD: Motion control ETD hook.

## Competing interests

The authors declare that they have no competing interests.

## Authors' contributions

BK was responsible for the design and implementation of the synergy controller on the Shadow Hand, data acquisition and analysis for all test subjects, and drafting of the manuscript. NK performed human hand motion data acquisition and analysis. EE conceived of the study and the synergy controller, participated in data acquisition for all limb absent subjects, and drafted major portions of the manuscript. All authors have read and approved the final manuscript.
